# Health information managers’ engagement in continuing professional development

**DOI:** 10.1177/18333583251378960

**Published:** 2025-09-29

**Authors:** Abbey Nexhip, Merilyn Riley, Kerin Robinson

**Affiliations:** 1 La Trobe University, Australia; 2 Goulburn Valley Health, Australia

**Keywords:** health information management, health information manager, health information management profession, health information management workforce, continuing professional development, professional development, professional competency, lifelong learning

## Abstract

**Background:**

Continuing professional development (CPD) involves ongoing learning to maintain and enhance professional competence. CPD for health information managers (HIMs) is embodied in the Health Information Management Association of Australia’s (HIMAA) Professional Competency Standards. The CPD engagement of Australia’s HIMs has not been explored.

**Objective:**

To examine HIMs’ engagement with professional body-initiated CPD, and the associated enablers and barriers.

**Method:**

A cross-sectional survey was administered to 72 Victorian graduate HIMs from four cohorts: 1985, 1995, 2005 and 2015. It elicited their perceptions of CPD and engagement with HIMAA events and activities.

**Results:**

When asked if engaging in CPD was important: 70.8% agreed; 9.7% disagreed; 19.4% were unsure. Motivations for engagement were networking, continuous learning and skill development and gaining insights from the work of others. Barriers to participation included movement outside of HIM roles, lack of time or interest and perceived irrelevance. The most common activities were special interest/working group(s) (27.8%) and other (sub)committees (18.1%). Seventy-two percent had attended an (inter)national conference or seminar/webinar. Notably, 57% had not participated in any HIMAA-related activities.

**Discussion:**

HIMs must continually build their knowledgebase and skills to align with the evolving health information ecosystem. A large proportion of participants acknowledged the importance of CPD; however, of concern, are the 29.1% who disagreed or were unsure, and the 57% who did not participate in any professional body-initiated CPD activities.

**Conclusion:**

There is room to strengthen HIMs’ engagement with CPD to ensure their commitment to career-centred lifelong learning, maintain professional competence by meeting a core competency outlined in the Professional Competency Standards and contribute to the continuing development of the profession.

**Implications for health information management practice:**

HIMAA should continue to diversify CPD content and formats to sustain and further enhance engagement, to ensure HIMs remain competent, and responsive to the changing health information environment.

## Introduction

In the ever-evolving, dynamic and complex field of health care, the importance of healthcare professionals maintaining currency with the latest developments in their field is paramount (Alshraa et al., 2024; Jiandani et al., 2015; Joynes et al., 2017). Friedman et al. (2000) defined continuing professional development (CPD) as “*the systematic maintenance, improvement and broadening of knowledge and skills, and the development of personal qualities necessary for the execution of professional and technical duties throughout the individual’s working life*” (p. 41). CPD underpins the requirement to remain current and ultimately maintain “fit[ness] to practi[c]e” (Power et al., 2011: 424). CPD is the lifelong process in which practitioners intentionally develop and maintain their knowledge, skills and attitudes, to ensure continuing professional competence and delivery of high-quality services (Batista et al., 2022; Jiandani et al., 2015; Main and Anderson, 2023). Engaging in CPD can also promote career advancement opportunities and strengthen one’s feelings of belonging and identity within a profession (Markova et al., 2013). This can be accomplished by engaging in formal activities such as seminars and conferences, studies towards formal qualifications, workshops, and reading journals and professional material (Jiandani et al., 2015), as well as informal activities such as peer collaboration, engagement in supervision, mentoring or coaching and reflective practice (Kumar, 2013). The modalities of CPD learning can be delivered in both online and physical formats. While emerging technologies are modernising CPD delivery in the healthcare sector, certain CPD activities, such as face-to-face lectures, practical workshops and seminars, remain essential (Byungura et al., 2022).

### Enablers and barriers to CPD engagement

There are many individual, organisational and systemic factors that can create enablers and/or barriers to participating in CPD. Engagement in CPD is encouraged with strong support from managers and colleagues, recognition of achievements and a workplace culture that values lifelong learning (Hakvoort et al., 2022; Haywood et al., 2012; Jeong et al., 2018). In contrast, poor leadership or limited organisational support structures can impede participation (Cameron and Masterson, 2001). Easily accessible (i.e. flexible delivery modes such as online learning), attractive and relevant topics, and user-friendly registration processes can increase participation (Campos-Zamora et al., 2022; Hanlon et al., 2021). Other enablers include opportunities for networking, and for knowledge and experience sharing (Jeong et al., 2018): personal motivation and commitment to professional excellence (Alshraa et al., 2024; Kurtović et al., 2024) and linking CPD with career progression (Kurtović et al., 2024; Vázquez-Calatayud et al., 2021). The major barriers include time and workload constraints (Hanlon et al., 2021; Summers, 2015), financial challenges through direct costs, conference registration fees, travel expenses or lost income due to absence from work, and lack of motivation or perceived reward of CPD involvement (Campos-Zamora et al., 2022; Kumar, 2013; Summers, 2015). Caring responsibilities and the need to balance work and personal life are also acknowledged as obstacles for CPD (Kumar, 2013).

### Regulatory frameworks and CPD requirements for health professionals in Australia

As CPD has evolved, it has transformed professional learning into a structured process, increasingly shaped and often mandated by professional bodies, with sanctions for non-compliance. It is now more planned and evaluated, aligning with the broader expectations of professionalism (Friedman, 2023). At the time of writing, 15 health professions in Australia were regulated under the *Health Practitioner Regulation National Law Act 2009* (as enacted by each state and territory in 2009–2010). This legislation establishes the National Registration and Accreditation Scheme, under which the Australian Health Practitioner Regulation Agency (AHPRA) operates (AHPRA, 2025). Registered health practitioners must undertake CPD to maintain their registration (AHPRA, 2025). CPD requirements vary: for example, 20 hours annually for professions such as occupational therapists (Occupational Therapy Board of Australia, 2019), podiatrists (Podiatry Board of Australia, 2015) and physiotherapists (Physiotherapy Board of Australia, 2015), 30 hours for psychologists (Psychology Board of Australia, 2015) and 60 hours for dental practitioners (Dental Board of Australia, 2015). These health professionals must maintain records of their learning goals, planned activities and reflections that demonstrate how CPD has improved or is expected to improve their practice, to ultimately achieve the main goal of health professionals: “protect[ing] patients and the general public” (Friedman, 2023: 591). These standards apply to the regulated professions. Health information management, like a number of other health professions in Australia, is not formally regulated under the National Registration and Accreditation Scheme.

### CPD landscape for health information managers

Continuous professional development is not mandated for health information managers (HIMs) under any national regulatory framework; however, it is strongly supported and guided by the peak professional body, the Health Information Management Association of Australia (HIMAA). The HIMAA (2023) HIM Professional Competency Standards serve as the primary framework underpinning HIMs’ CPD, positioning lifelong learning as a core responsibility throughout entry-level, intermediate and advanced stages of the professional continuum. According to Friedman (2012), embedding CPD within governance structures signifies a level of maturity and commitment to maintaining high professional standards. This perspective aligns with HIMAA’s approach, as its CPD program is embedded within its governance framework. The emphasis on CPD begins during tertiary education, where students enrolled in HIMAA-accredited university degrees actively engage in CPD activities such as reflective practice. The embedding of lifelong learning in the curriculum equips students with the tools and skills necessary to engage in professional development throughout their careers. Further guidance is provided by the HIMAA (2015) Professional Practice Guidelines, which outline various membership obligations, including those relevant to CPD, such as “advancing health information management knowledge and quality practice through continuing participation in education, research, publications, presentations and interdisciplinary collaboration” (p. 7). HIMs may voluntarily elect to become members of HIMAA, which actively promotes CPD participation through its Professional Credentialling Program. This program requires HIMs to accrue 100 CPD points per financial year cycle to attain “credentialling,” a process for validating professional currency. Points may be earned through a range of activities relevant to the health information management profession, including but not limited to attending conferences, seminars, workshops and webinars; completing short courses and other educational activities; engaging in research and/or publication; and contributing to professional education and/or service to the profession (HIMAA, 2025). Together, these guidelines shape the structured, profession-led approach to CPD for HIMs in Australia, with the HIMAA Professional Competency Standards serving as the overarching framework for maintaining and advancing professional practice.

While there have been several studies on the CPD participation of other health professionals (Batista et al., 2022; Gould et al., 2007; Hakvoort et al., 2022; Hanlon et al., 2021; Hobbs et al., 2021; Jeong et al., 2018; Negussie et al., 2024; Power et al., 2011; Stewart et al., 2020; Vázquez-Calatayud et al., 2021; Williams and Edlington, 2019), there is no discoverable research relating to CPD participation by HIMs in Australia. This study offers insights that address this gap in the literature.

### Aims and objectives

This research was part of a larger study that aimed to investigate the motivators of HIMs in the construction of their professional identity. Job satisfaction, engagement with the profession and perceptions of career success were also analysed separately to motivation and professional identity. The objectives of this component of the research were to

(1) Determine HIMs’ engagement with professional body-initiated CPD; and,
(2) Identify the enablers of, and barriers to, their engagement.

## Method

### Study design

This study employed a convergent mixed-methods approach within a cross-sectional study design, as described by Creswell and Creswell (2017).

### Sample

Data were collected, in July 2021, from the 1985, 1995, 2005 and 2015 graduate cohorts of the medical record administration and health information management degrees at La Trobe University (LTU) and Lincoln Institute of Health Sciences, Victoria, Australia. The method is detailed in Nexhip et al. (2024, 2025). Participants were recruited via three channels: a database maintained by LTU’s Discipline of Health Information Management, LinkedIn and the researchers’ professional networks. Graduates who had retired or transitioned into careers outside health information management were included. The exclusion criteria were graduates who were deceased, untraceable or members of the research team. Following application of these criteria, 99 out of 136 eligible graduates were included in the study.

### Data collection

Data were collected via a purposively designed, online survey, developed using Research Electronic Data Capture. The survey comprised closed and open-ended items designed to gather information on graduates’ motivation, professional engagement, job satisfaction, professional identity and career reflections. The closed questions relevant to this component of the study included multiple-choice items on involvement in HIMAA activities and attendance at related events, and a question on the participants’ perceived importance of professional development and associated engagement. The open-ended questions invited participants to describe why undertaking professional development and/or engagement was important to them.

### Data analysis

Quantitative and qualitative data were analysed using Microsoft Excel. Frequency counts and descriptive statistics (quantitative) were generated to summarise participant characteristics and responses to closed questions. Qualitative data were analysed using conventional content analysis, through application of Hsieh and Shannon’s (2005) approach, in which categories are derived from the data without reference to pre-existing frameworks. Responses were reviewed to identify meaningful categories, and relevant quotations were assigned to each category.

### Ethics approval

The study was approved by the LTU Human Research Ethics Committee (No. 21167).

## Results

### Response rate

As reported previously by Nexhip et al. (2024, 2025), 72 responses were received from 99 invitations to complete the survey, representing a response rate of 72.7%.

### Perceived (un)importance of CPD and engagement

**Figure 1.**
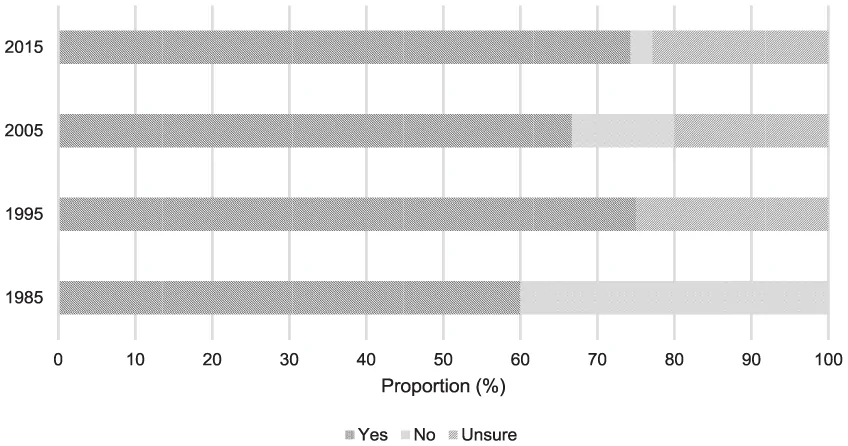
Proportion of participants’ perceptions of the importance of CPD and/or engagement with CPD, by cohort. CPD: continuing professional development.

Figure 1 outlines the responses to the question “*In general, is participating in health information management professional development and/or engagement . . . important to you?*”

The 1995 and 2015 cohorts had the highest proportion of “Yes” responses (75% and 74.3% respectively). The largest proportion of respondents who were “Unsure” were also from the 1995 cohort (25%); 40% from the 1985 cohort indicated that they did not deem CPD important. Overall, 70.8% of the 72 respondents (*n* = 51) reported that CPD engagement was important, 19.4% (*n* = 14) were unsure and 9.7% (*n* = 7) said it was not important.

Table 1 outlines the key motivations for engaging in CPD and professional association activities. A total of 45/72 responses (62.5%) were received for the question: “*Why is it important to you to undertake health information management professional development and/or engagement?*” The most frequently reported factor was the value of maintaining relationships within the profession (*n* = 23), closely followed by the desire for ongoing learning, professional growth and skill enhancement (*n* = 22), and an interest in remaining informed about changes in the profession (*n* = 22). Respondents also valued the learning opportunities about initiatives, projects and practices being undertaken by peers in other organisations (*n* = 8). Three respondents referenced the need to stay current with technological and clinical advancements.

**Table 1.** Qualitative responses indicating why professional development is important.

Category	*n*	Qualitative responses
Networking and connectedness with peers	23	“It is important to build relationships” [77] “To stay connected with fellow HIMs” [61] “It is important to maintain contact with HIMs across the industry” [50] “Networking opportunities that it brings” [25] “Keeping [in] contact with my professional colleagues” [24] “Meet up with colleagues from past workplaces and meet new HIMs” [9] “Network with peers” [4]
Continuous learning, professional growth and skills development	22	“Understand the gaps in one’s knowledge and address these through further learning” [82] “To grow and evolve as a HIM. . .” [43] “Continuous professional growth and development” [72] “There is always something new to learn in HIM career” [47] “Top up and/or learn new information to assist me in my day to day [work]” [83] “Expand the knowledge of the organisation at which I work” [79] “These PD engagements are important because we acquire knowledge” [41]
Keep abreast with changes within the profession	22	“Primarily in order to stay afloat with the trajectory of how the profession is evolving” [17] “To learn about the changes in the industry” [12] “I think it is a fantastic opportunity to network and stay up to date with the many types of careers available to HIMs” [27] “What is happening in the field more generally” [51] “It is important to me to stay in touch with the profession and what is happening because I work in a non-traditional HIM role and it’s easy to feel like I’m out of the professional loop” [2] “It is important to keep abreast and support upcoming professionals in the industry” [48] “Keeping abreast of changes within the profession” [14]
Insights into the work of other organisations	8	“Learn about what others are working towards” [14] “It’s important to learn about projects and innovating work undertaken by your peers” [20] “To see what other HIMs/HIS departments have been involved in” [30] “[F]ind out how other health services approach current issues” [9] “Keep up to date with what other organisations are doing” [4]
Remain up to date with new health technologies/medical practices	3	“To learn about the new technology” [12] “Keeping up to date with new ideas and technology” [24] “[T]o keep up with developments in technology, medical/surgical practices and apply knowledge to coding process” [9]

*Note*: Individual responses could be assigned to multiple categories. Therefore, the total number of category assignments (*n* = 78) exceeds the total number of responses (*n* = 45).

There were six responses to the question: “*Why do you consider health information management and/or engagement to be unimportant?*” The most common reasons cited were a transition out of the health information management profession or not currently working in a “traditional” HIM role, a colloquial and non-defined reference which the researchers have interpreted, for the purpose of this research and in the absence of clarification by the respondents, as a hospital-based Health Information Service position. One respondent explained that they had “learn[t] enough on the job not to have sought out further development” [16], while another noted they “purely don’t have time or interest” [81]. One respondent reported reluctance to invest in CPD activities, commenting that they “would never spend money on attending any of these events [as they are not] useful to my job as a coder,” although they acknowledged that they “would be inclined to attend these events” [31] if working in a managerial role. Another respondent highlighted a perceived lack of relevance, noting that CPD activities “have not always been relevant to my roles” [48].

### Involvement in national association committees/groups and professional events

Participation in HIMAA’s Special Interest or Working Groups (Table 2) represented the highest proportion of engagement among all HIMAA CPD activities, with an overall rate of 27.8%. The highest participation rate was reported by the 2005 cohort (40%). Overall participation in State Branch Executives was 9.7%, and 18.1% for other (sub)committees. Engagement in these activities was the highest in the 1995 cohort, at 16.7% and 25% respectively. A few graduates participated in the Professional Credentialling Scheme (5.6%) or the Editorial Board for the *Health Information Management Journal (HIMJ)* or the *HIM-Interchange (HIM-I)* Subcommittee (2.8%). No participants reported membership of the Board of Directors. Across all cohorts, a majority of the participants (57%) reported having had no involvement in any HIMAA activities.

**Table 2.** Number and proportion* of participations in national and international association-related activities and events, by cohort.

Activity/event	1985		1995		2005		2015		Total	
	*n =* 10	%	*n =* 12	%	*n =* 15	%	*n =* 35	%	*n =* 72	%
Activity										
Special interest or working groups	2	20	4	33.3	6	40	8	22.9	20	27.8
Professional credentialling scheme	1	10	0	0	1	6.7	2	5.7	4	5.6
Editorial board (*HIMJ)* or its *HIM-I* subcommittee	1	10	0	0	0	0	1	2.9	2	2.8
Board of directors	0	0	0	0	0	0	0	0	0	0
State branch executive	1	10	2	16.7	0	0	4	11.4	7	9.7
Other (sub)committees	2	20	3	25	2	13.3	6	17.1	13	18.1
Other	1	10	1	8.3	1	6.7	0	0	3	4.2
None of the above	6	60	6	50	8	53.3	21	60	41	57
(Inter)national event(s)										
HIMAA National Conference	7	70	9	75	9	60	18	51.4	43	59.7
HIMAA seminar/webinar	5	50	8	66.7	7	46.7	15	42.9	35	48.6
International Federation of Health Information Management Congress	0	0	2	16.7	0	0	0	0	2	2.8
Have not attended any of the above	3	30	2	16.7	4	26.7	11	31.4	20	27.8

* Proportion is calculated out of the total number of graduates from the relevant cohort.

## Discussion

This study provides new insights into the enablers and barriers that shape CPD engagement and professional association involvement among a cross-section of Victorian health information management graduates.

### Patterns of CPD engagement

While over 70% of participants (*n* = 51) across all cohorts affirmed the importance of CPD, actual engagement in HIMAA activities was limited, with 57% reporting no involvement. This attitudinal-behavioural disconnect may reflect a broader global trend observed in health professionals’ literature, where recognition of the value of CPD does not always translate into engaging in these activities. For instance, Stewart et al. (2020) reported that, among 205 United Kingdom-registered physiotherapists, 92% valued CPD, yet 43% still regarded it “as a chore” (p. 6). Negussie et al. (2024) also found that although 76.8% of 228 Ethiopian medical laboratory professionals believed CPD was important for their careers, 17.1% had no comments on CPD and 6.1% considered it less important – figures that closely align with the 19.4% of respondents in the present study who were unsure about the importance of CPD and the 9.7% who deemed it unimportant.

In this study, engagement was most evident in Special Interest or Working Groups (27.8%), particularly among mid-career professionals from the 2005 cohort (40%). Interestingly, the same cohort experienced the highest levels of professional stress (53.3%), reported in Nexhip et al.’s (2024) study involving the same sample. Nexhip et al. (2024) suggested that this stress might be attributed to insufficient upskilling, among other factors. The current finding, however, complicates that interpretation: rather than indicating a lack of engagement, it is feasible that mid-career professionals are, in fact, more actively participating in professional groups than the other cohorts, to enhance their confidence and competence. Paradoxically, this involvement may be contributing to increased stress due to the added responsibilities it brings to their workload. This interpretation is supported by Welch et al. (2019) who highlighted mid-career as a peak period for burnout and diminished professional vitality, attributed to factors such as competing professional and personal demands, and stagnation in career progression.

The activity in which the second highest proportion of respondents engaged was other (sub)committees (18.1%). While the survey did not capture the specific nature of these committees, it is probable that they included roles in advocacy, education or professional activity/event planning – suggesting that some HIMs contribute to the profession through more behind-the-scenes, invitational and temporary forms of involvement. Minimal engagement was observed in roles such as State Branch Executives and the Editorial Board (9.7% and 2.8% respectively), and no participants from this sample had held a position on the Board of Directors. This may reflect the significant time commitment and higher level of specialised knowledge, skills and professional experience these positions typically require, in comparison to other (sub)committee roles that might not demand such expertise or time dedication. It could also be a wider reflection of the general view that professional associations often struggle to recruit members in a volunteer capacity. Wang and Ki (2018), in their study of 120,540 members across 18 American professional associations, including those for nurses, project managers and accountants, found that despite members’ general agreement with the association’s values, engagement often remained low unless individuals perceived a clear personal or professional benefit. Similar trends were reflected in the Australian Bureau of Statistics’ (2021) General Social Survey, which showed a widespread decline in the proportion of people involved in volunteering roles over time. Taking this into consideration, CPD uptake was more prominent in one-off activities that do not require sustained voluntary involvement; for example, 72.2% of respondents reported attending at least one (inter)national conference, seminar or webinar. This preference for low repeated commitment may reflect a broader tendency among HIMs to favour events or activities that are time-efficient and easier to integrate into schedules. As observed in other healthcare professions, time constraints are one of the most repeatedly cited barriers to ongoing CPD engagement (Hanlon et al., 2021; Hobbs et al., 2021; Jeong et al., 2018; Schostak et al., 2010; Stewart et al., 2020; Summers, 2015). One-off events not only reduce the burden of regular commitment but may also offer enhanced networking opportunities and exposure to peer practices – factors frequently cited by participants in this study as key motivators to engaging in CPD.

### Generational trends

Cohort analysis revealed further nuances, specifically that the 1995 and 2015 cohorts showed the highest belief in the importance of CPD (75% and 74.3% respectively), while the 1995 cohort also had the largest proportion of respondents who were unsure (25%). Notably, 40% of the 1985 cohort also indicated that they did not deem CPD to be important. This aligns with Wray et al.’s (2009) findings, from a cohort of nurses, that those aged over 50 participated in fewer CPD activities than their younger peers. This polarisation within older cohorts may be shaped by varying experiences of the evolving profession (including HIMAA’s development), shifting workplace expectations, reduced perceived relevance, proximity to retirement or divergent career trajectories. It may also reflect generational attitudes towards formalised learning, where senior practitioners may feel less need to formally credential their expertise or see limited return on CPD investment late in their careers (Wang and Ki, 2018; Wray et al., 2009). This may also indicate the influence of career progression as a motivator of CPD participation, as engagement is often heightened when individuals are actively pursuing career advancement (Bolam et al., 2005; Kurtović et al., 2024; Vázquez-Calatayud et al., 2021). For more senior professionals, it is possible that they have already achieved many of their career goals, or as Gould et al. (2007) described, reached a “ceiling” (p. 607), making further advancement less of a priority. As a result, they may view CPD as unnecessary for their current professional stage. Additionally, some graduates may have transitioned into different fields, perceiving HIMAA-endorsed activities as irrelevant. It is important to note the relatively small sample size of 1985 graduates compared to other cohorts. Collectively, these findings suggest that while health professionals generally support the concept of CPD, significant barriers may impede the translation of positive attitudes into meaningful participation.

### Enablers and barriers to engagement

Qualitative responses reinforced the multifaceted nature of CPD engagement. Intrinsic drivers included networking and peer connectedness, continuous learning and skills development, and staying abreast of changes within the profession. Participants also valued exposure to the work of other organisations and new technologies, although these were mentioned less frequently. Networking, in particular, has consistently been identified in the literature as a major driver for participation in CPD. Over time, CPD has evolved from a focus on formal activities (i.e. training sessions and events) to also include informal activities like networking, socialising and reflective learning (Friedman, 2023). HIMAA at the time of writing this paper supports this shift by providing both formal and informal CPD opportunities. In a non-health context, a report commissioned by the United Kingdom Department for Education and Skills and authored by Bolam et al. (2005) emphasised that CPD fosters professional dialogue, collaboration and a sense of community. Within health care, Stewart et al. (2020) found that peer connection enhanced motivation to engage in CPD, while Markova et al. (2013) noted that networking opportunities and targeted offerings shaped positive perceptions and uptake. In this context, networking is an essential practice to ensure individuals sustain professional relevance and remain up to date with the advancements within the sector. This aligns with International Federation of Health Information Management Association’s (2022) assertion that the health information management profession must iteratively adapt to industry changes, to effectively support the healthcare system in the relevant countries.

Despite these enablers, barriers persist. While most participants recognised the value of CPD, a concerning proportion of respondents expressed ambivalence (19.4%, *n* = 14) or disapproval (9.7%, *n* = 7) of the importance of CPD. Among those who elaborated, common reasons for this were the perceived lack of relevance of CPD activities to their current role(s), scarce time or interest and the belief that on-the-job experience was adequate. These obstructions are consistent with the broader CPD literature. Time and workload are regularly cited deterrents to engaging in CPD, across many industries including health (Hanlon et al., 2021; Hobbs et al., 2021; Jeong et al., 2018; Schostak et al., 2010; Stewart et al., 2020; Summers, 2015), education (Dheeraj and Kumari, 2024; Broad, 2015) and engineering (Geronimo et al., 2024). Stewart et al. (2020) and Hobbs et al. (2021) also found that perceived irrelevance of CPD offerings hindered participation among paramedics and physiotherapists. This highlights a universal truth: for CPD to be effective, regardless of professional context, it must be both relevant and accessible.

### Voluntary participation

Another consideration is the voluntary nature of CPD participation. While mandatory models can drive registration, they risk superficial participation. For example, Williams and Edlington (2019) found that many paramedics completed CPD solely to maintain registration, limiting meaningful engagement aligned with their professional needs. Voluntary models, on the other hand, allowed for genuine-interest participation, rather than simply a “tick-box” exercise (New South Wales Productivity Commission, 2022). Stewart et al. (2020) noted similar outcomes, where CPD driven by personal desire as opposed to enforced need was deemed to be more satisfying. Such models, however, rely heavily on individual motivation and other enablers of CPD engagement such as organisational support. Professional associations play a critical role in supporting CPD uptake. Wang and Ki’s (2018) survey, as described earlier, identified several key enablers to member engagement: the need for fulfilment, perceived organisational support and favourable attitudes towards the association. Members who believed that their professional needs were being met were more likely to participate in association-led initiatives such as volunteering. Similarly, Ki and Hon (2007) found that positive stances on an organisation significantly influenced individuals’ behaviour. Within the health information management context, this may suggest that HIMAA and similar organisations can further enhance engagement by building their existing value through varied CPD offerings, clear communication and tangible member benefits (e.g. discounts, professional networking opportunities, and accessible and flexible CPD programs; Markova et al., 2013).

Importantly, the study found that while 57% of participants did not engage in formal professional association activities, the remaining 43% did engage. This suggests that lack of engagement may not be a matter of disinterest, but rather a reflection of the discussed barriers such as time, competing priorities or a perception that some activities did not align with their professional needs. The health information management workforce spans many contexts – such as public and private hospitals, clinical quality registries, government health departments, health technology companies and community health centres – rendering it uniquely positioned within the healthcare system. CPD programs must reflect this complexity through matched diverse, inclusive and practice-relevant CPD offerings, and motivate their membership to engage in such initiatives.

### Limitations

This study has several limitations. The use of branching logic in the survey’s open-ended questions limited the researchers’ ability to fully capture why some participants did not engage in CPD, thereby restricting insight into barriers to participation. Data were collected during the COVID-19 pandemic, a period that disrupted professional practices, workplace interactions and access to CPD. These responses may not fully reflect the current, post-pandemic realities as HIMs’ attitudes and engagement patterns may have shifted. The cross-sectional design offers only a snapshot in time, without accounting for changes over an individual’s career span. Additionally, the questions in the study primarily focused on HIMs’ engagement with profession-initiated CPD. Exploring CPD participation at the organisational level or through alternative avenues was outside the scope of the study. The sample, drawn from LTU and Lincoln Institute alumni, may also limit generalisability to the broader Australian HIM workforce. Nexhip et al. (2024, 2025) have previously reported additional limitations. Despite these limitations, the findings have important implications.

## Conclusion

This study contributes new evidence on Australian HIMs’ engagement with CPD. The majority of HIMs recognised the value of CPD for maintaining professional currency, expanding expertise, strengthening peer networks and remaining abreast of industry developments. Identified barriers, such as time constraints and perceived irrelevance of CPD initiatives, limited participation. Given the profession’s diverse roles across the healthcare sector, CPD offerings must remain varied to ensure perceived relevance and as a corollary, increased participation. Further research is warranted to explore CPD participation across specific content areas and delivery modes to better understand what drives engagement. Replicating this study with a broader sample would enhance the generalisability of findings to inform more targeted, strategic improvements to CPD practices within the health information management profession. Potentially, these strategies would not only support individuals’ professional development and by extension, their contribution to their employer organisation’s growth, but also strengthen the profession’s overall capacity to meet the evolving demands of the Australian healthcare system. Sustained CPD is not only an individual responsibility, but a collective imperative that is crucial for ensuring that the health information management workforce remains responsive, relevant and agile to the healthcare’s system dynamicity.
